# Streptomycin Induced Stress Response in *Salmonella enterica* Serovar Typhimurium Shows Distinct Colony Scatter Signature

**DOI:** 10.1371/journal.pone.0135035

**Published:** 2015-08-07

**Authors:** Atul K. Singh, Rishi Drolia, Xingjian Bai, Arun K. Bhunia

**Affiliations:** 1 Molecular Food Microbiology Laboratory, Department of Food Science, Purdue University, West Lafayette, Indiana, United States of America; 2 Department of Comparative Pathobiology, Purdue University, West Lafayette, Indiana, United States of America; KIIT University, INDIA

## Abstract

We investigated the streptomycin-induced stress response in *Salmonella enterica* serovars with a laser optical sensor, BARDOT (**ba**cterial **r**apid **d**etection using **o**ptical scattering **t**echnology). Initially, the top 20 *S*. *enterica* serovars were screened for their response to streptomycin at 100 μg/mL. All, but four *S*. *enterica* serovars were resistant to streptomycin. The MIC of streptomycin-sensitive serovars (Enteritidis, Muenchen, Mississippi, and Schwarzengrund) varied from 12.5 to 50 μg/mL, while streptomycin-resistant serovar (Typhimurium) from 125–250 μg/mL. Two streptomycin-sensitive serovars (Enteritidis and Mississippi) were grown on brain heart infusion (BHI) agar plates containing sub-inhibitory concentration of streptomycin (1.25–5 μg/mL) and a streptomycin-resistant serovar (Typhimurium) was grown on BHI containing 25–50 μg/mL of streptomycin and the colonies (1.2 ± 0.1 mm diameter) were scanned using BARDOT. Data show substantial qualitative and quantitative differences in the colony scatter patterns of *Salmonella* grown in the presence of streptomycin than the colonies grown in absence of antibiotic. Mass-spectrometry identified overexpression of chaperonin GroEL, which possibly contributed to the observed differences in the colony scatter patterns. Quantitative RT-PCR and immunoassay confirmed streptomycin-induced GroEL expression while, aminoglycoside adenylyltransferase (*aadA*), aminoglycoside efflux pump (*aep*), multidrug resistance subunit *acrA*, and ribosomal protein S12 (*rpsL*), involved in streptomycin resistance, were unaltered. The study highlights suitability of the BARDOT as a non-invasive, label-free tool for investigating stress response in *Salmonella* in conjunction with the molecular and immunoassay methods.

## Introduction

*Salmonella enterica* is responsible for gastrointestinal diseases and is a major foodborne pathogen of worldwide concern [1]. It is also one of the top five foodborne pathogens (the other four being species belonging to *Campylobacter*, *Clostridium*, *Staphylococcus*, and *Norovirus*) in the United States, and is the leading cause of hospitalization (35%) and deaths (28%) resulting in about 1 million cases of illness and 378 deaths annually [2,3]. According to the National Enteric Disease Surveillance: *Salmonella* Annual Report, 2011 [4], the two most common serovars *i*.*e*., *Salmonella enterica* subsp. *enterica* serovar Typhimurium (*S*. Typhimurium) and *Salmonella enterica* subsp. *enterica* serovar Enteritidis (*S*. Enteritidis) [5] are responsible for 41.5% of the total outbreaks reported among the top 20 human-origin *Salmonella* serovars. Also, these two serovars represent about 60% of the total *Salmonella*-related outbreaks globally [1].

With the extensive and widespread application of antibiotic as a therapeutic agent in animals and humans, and as a growth promoter in livestock, bacteria have been exposed to sub-inhibitory (non-lethal) dose of antibiotics. This has played critical role in the evolution of antibiotic resistance [6] and selection of antibiotic resistant bacteria [7]. In recent years, the widespread and often indiscriminate use of antibiotics worldwide has resulted in the emergence of multidrug-resistant (MDR) bacterial pathogens, such as extended-spectrum β-lactamase (ESBL) Gram-negative bacteria [8,9]. Such a significant emergence of antibiotic resistance in the bacterial pathogens especially in *Enterobacteriaceae* family has become a global concern posing a major threat to the public health and livestock [10–12]. Antibiotic resistance is generally acquired due to (i) enzyme modification and hydrolysis of antibiotics, (ii) reduced uptake by the cells, (iii) increased efflux, (iv) alteration or production of new target site(s), and/or (v) over-expression of drug target(s) [13]. Transfer of antibiotic resistance between commensal and pathogenic members of the *Enterobacteriaceae* family has been reported [14]. *S*. Typhimurium, being a member of the *Enterobacteriaceae* family, may acquire antibiotic resistance genes through horizontal gene transfer from other bacteria and/or natural environment [15] to develop resistance to multiple antibiotics [16]. Chen et al [17] isolated multiple-drug-resistant *S*. Typhimurium strains from retail meat; out of the 133 isolates, 73 strains were resistant to streptomycin indicating a widespread distribution of streptomycin resistance in *Salmonella* isolates.

Although streptomycin, an aminoglycoside is not used as a therapeutic agent for *Salmonella* infection, streptomycin resistance has been widely used as an epidemiological marker. Resistance to streptomycin is analogous to the phenotypic characteristic observed for multidrug resistance to ampicillin, chloramphenicol, streptomycin, sulfonamides and tetracyclines in *S*. Typhimurium DT104 [18]. The effect of antibiotics on *Salmonella* spp. has been studied using suspension cultures by employing conventional and molecular techniques [19,20]. However, in the physical world, bacterial pathogens show multicellular behavior in the colonial form [21,22] (e.g., as biofilms, small colony variants, and persisters) on the surfaces of food, oral cavity, hospital settings, and in various environmental niches. To demonstrate insights of colony development, time-lapse fluorescence study has revealed continued binary fission of single bacterial cell expanding to form colony first in two-dimensional space and consequently into a three-dimensional arrangement [23]. Furthermore, antimicrobial-induced stress may also affect bacterial virulence and biofilm formation ability [24]. Therefore, our interest was to study the effect of antibiotic on bacterial colony using a laser sensor, BARDOT (**ba**cterial **r**apid **d**etection using **o**ptical scattering **t**echnology) that generates visual scatter signature of colony, and at the same time retains the integrity and viability of colony for further physiochemical, molecular and immunological characterizations.

In BARDOT, a laser beam (635 nm, 1 mW) impinges in the center of bacterial colony growing on Petri-dish producing scatter signature that can be used for differential interrogation of bacteria at the genus, species and serovar levels [25]. BARDOT represents a unique merger of a modern day label-free laser-based sensing tool and an age-old microbial isolation and purification device, Petri-dish, invented by Julius Richard Petri in 1887 [26]. We have successfully used BARDOT for detection and identification of *Listeria* species [25,27], *Vibrio* spp. [28], *S*. *enterica* serovars [29], Shiga-toxigenic *Escherichia coli* [30], *Campylobacter* spp. [31], and *Bacillus* spp. [32,33]. Therefore, the objective of this study was to investigate and understand the effect of antibiotics, especially on the colonial form of antibiotic resistant strains of pathogenic bacteria under *in vitro* condition using BARDOT and complementary molecular methods.

## Results and Discussion

**Fig 1:**
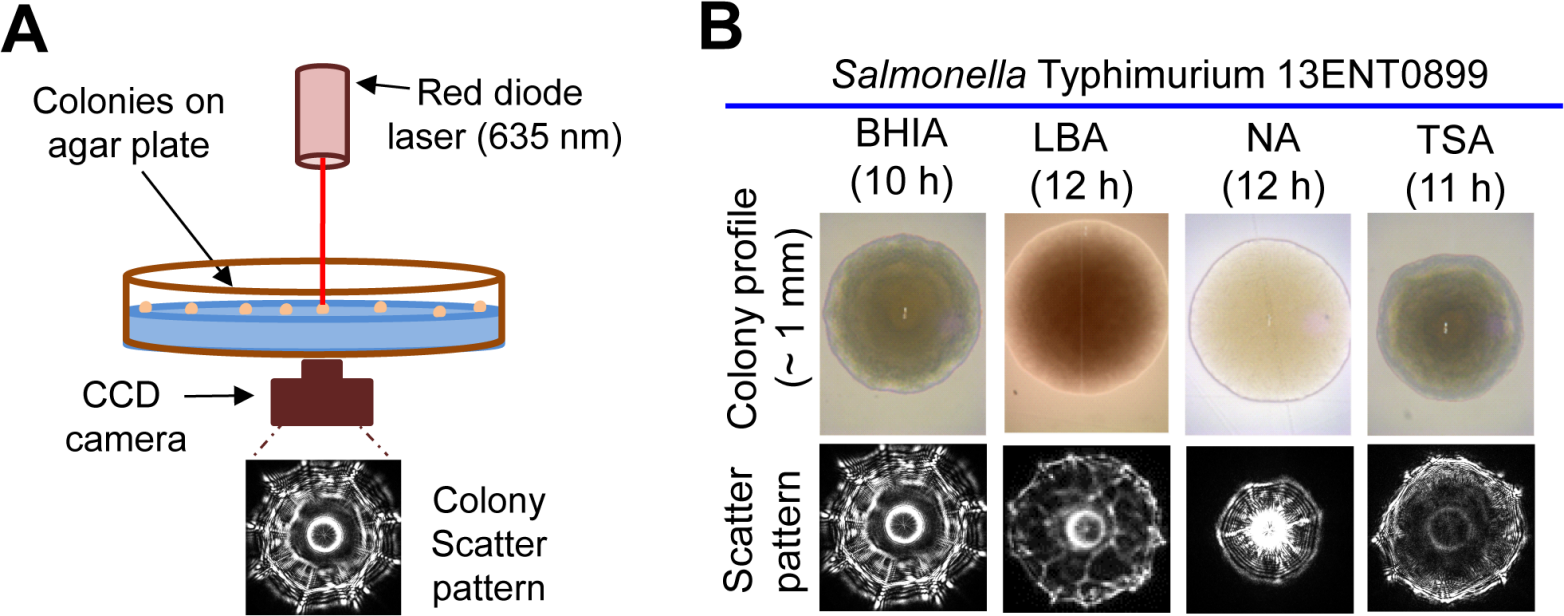
Schematic for capturing scatter pattern of colony with optical sensor and selection of optimal media. **(A)** Schematics of BARDOT to acquire scatter patterns of colonies. **(B)** Evaluation of different non-selective agar media to capture scatter pattern of *Salmonella enterica* serovar Typhimurium 13ENT0899 colonies. Brain–heart infusion agar (BHIA); Luria–Bertani agar (LBA); Nutrient agar (NA); Tryptic soy agar (TSA). Scatter patterns were captured when the colony diameter reached to 1.2 ± 0.1 mm after 10–12 h of incubation.

The goal of this study was to investigate antibiotic-induced stress response in pathogens using BARDOT by monitoring changes in the colony scatter patterns (**Fig 1A and S1 Video**). Streptomycin was used as a model antibiotic since *Salmonella* spp. are generally resistant and show high tolerance for this antibiotic [17,34,35].

### Optimal growth media to study the effect of antibiotic resistance by BARDOT

For the best application of optical biosensor, and to study the antibiotic-induced stress-response in bacterial colony, it is important to find a suitable agar medium without any inhibitory/selective compound. Optimal medium for *S*. Typhimurium will generate colony scatter patterns with maximum differentiating features such as Zernike moment (spokes and rings) and Haralick texture (granularity of image background) [29]. The selective differential medium, xylose lysine tergitol-4 (XLT4) proposed in our earlier study [29] was not used since it contains inhibitory agents that may impart inherent effect on scatter pattern, and may compromise any changes in scatter patterns caused by the streptomycin. Therefore, we tested several non-selective agar media including the brain-heart infusion agar (BHIA), Luria–Bertani agar (LBA), nutrient agar (NA), and tryptic soy agar (TSA) for this purpose. Human isolates of *S*. Typhimurium strains (13ENT1277, 13ENT1140, 13ENT1288, and 13ENT0899) obtained from the Indiana State Department of Health (Indianapolis, IN) were used as model pathogen. A total of 240 scatter images were generated from two independent experiments (60 images/media), to find the optimal media for BARDOT. We found that one of the test strains of *S*. Typhimurium (13ENT0899) grown on BHIA produced multiple colony scatter features (spokes and rings) which were distinct from that of colonies grown on LBA, NA, or TSA (**Fig 1B**). It was not unexpected since our previous studies have shown the media-dependent variations in the scatter pattern of bacterial colony in *S*. *enterica* serovars [29], *Bacillus* spp. [33]; and *E*. *coli* [30,36]. Thus, BHIA was selected for all future experiments. All cultures were grown on BHIA and 250 scatter patterns were collected from two independent experiments (60 images/strain) to build the *S*. Typhimurium (ST) scatter image library. This ST-library was used to analyze qualitative and quantitative differences in the scatter patterns of *S*. Typhimurium colonies grown in the presence or absence of streptomycin.

### MIC of streptomycin

**Fig 2:**
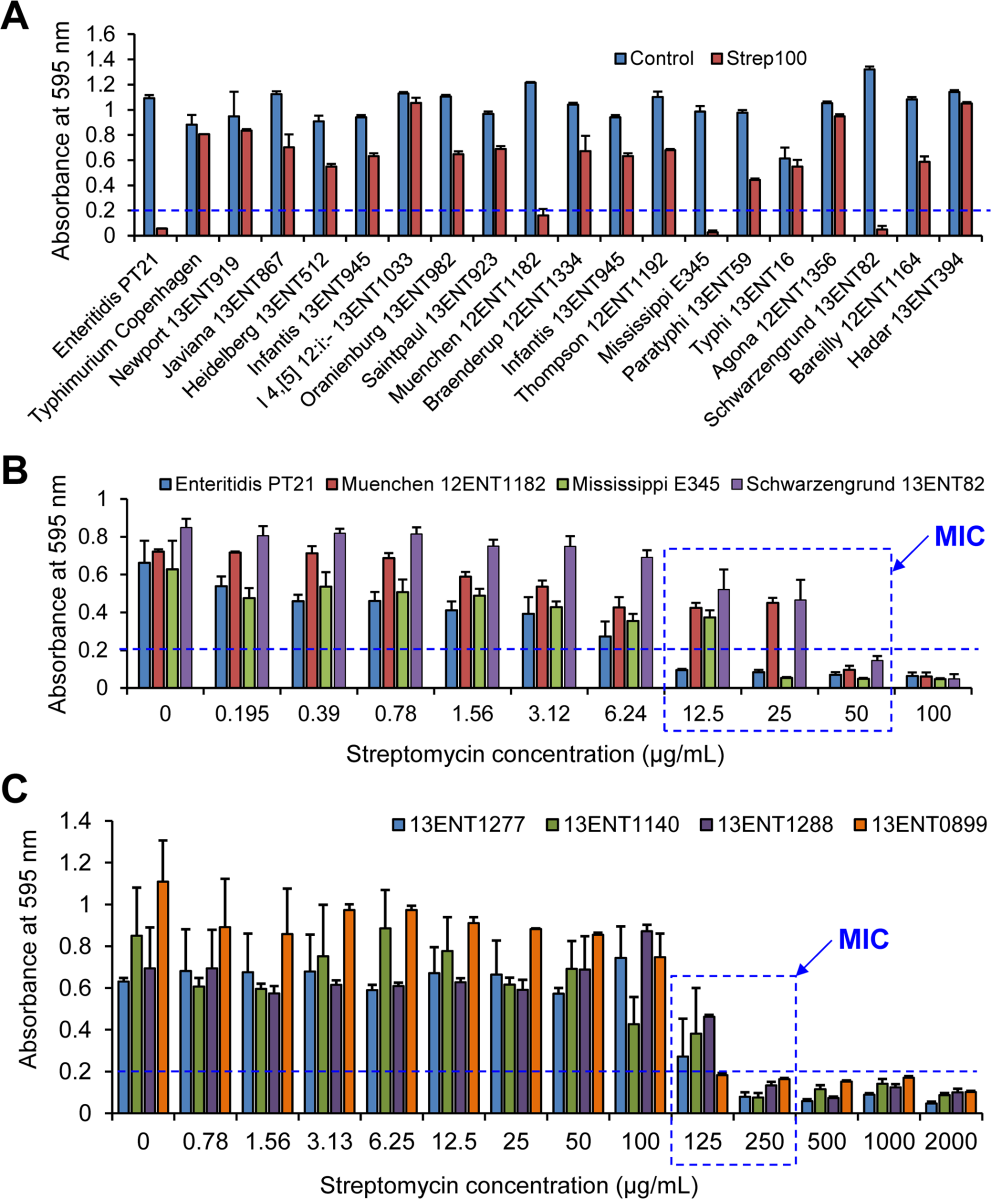
Minimum inhibitory concentration (MIC) of streptomycin against top 20 *Salmonella enterica* serovars. **(A)** Effect of streptomycin at 100 μg/mL (Strep100) on top 20 human-origin *S*. *enterica* serovars arranged in the order of outbreak incidence as per the National *Salmonella* Surveillance Annual Report, 2009. (**B**) Analysis of MIC of the streptomycin-sensitive (<100 μg/mL) *S*. *enterica* serovars. Absorbance of blank (LB broth) was 0.109 ± 0.052. (**C**) MIC of streptomycin-resistant *S*. enterica serovars Typhimurium (13ENT1277, 13ENT1140, 13ENT1288, and 13ENT0899). Broken straight line indicate threshold absorbance value (0.2), and an absorbance > 0.2 indicated growth of cultures in the wells of micro-titer plate.

Initially, the top 20 human-origin *S*. *enterica* serovars were tested against the streptomycin at a concentration of 100 μg/mL, and all but four serovars; *S*. Enteritidis PT21, Muenchen 12ENT1182, Mississippi E345, and Schwarzengrund 13ENT82 were found resistant (**Fig 2A**). The minimum inhibitory concentration (MIC) was analyzed by using micro-titer plate broth dilution method after taking spectrophotometric absorbance measurements at 595 nm [37]. The MIC of Enteritidis PT21 and Mississippi E345, was 12.5 and 25 μg/mL, respectively; while MIC for both Muenchen 12ENT1182, and Schwarzengrund 13ENT82 was 50 μg/mL (**Fig 2B**). None of the *S*. Typhimurium serovars from our collection was sensitive to this concentration. Select four strains of *S*. Typhimurium strains (13ENT1277, 13ENT1140, 13ENT1288, 13ENT0899) were further examined for their response to a streptomycin concentration of higher than 100 μg/ml and the MIC for strains 13ENT1277, 13ENT1140, and 13ENT1288 was 250 μg/mL, and for the strain 13ENT0899, it was 125 μg/mL (**Fig 2C**). These MIC values of streptomycin coincides with the earlier MIC values (48–256 μg/mL) reported for 51 *S*. Typhimurium isolates of nosocomial origin with multiple-drug resistance traits [34]. Based on these results, in all future experiments, we used sub-inhibitory concentrations of streptomycin at 1.25 μg/mL to 5 μg/mL for streptomycin-sensitive serovars; and 25 μg/mL and 50 μg/mL for streptomycin-resistant serovars to visualize their effect on the optical scattering properties of the colonies. A concentration of 100 μg/mL of streptomycin was not selected for streptomycin-resistant serovar, since it affected the *S*. Typhimurium growth taking more than 24 h to reach the desired colony size (~1 mm) for BARDOT analysis.

### Effect of streptomycin on signature scatter pattern

**Fig 3:**
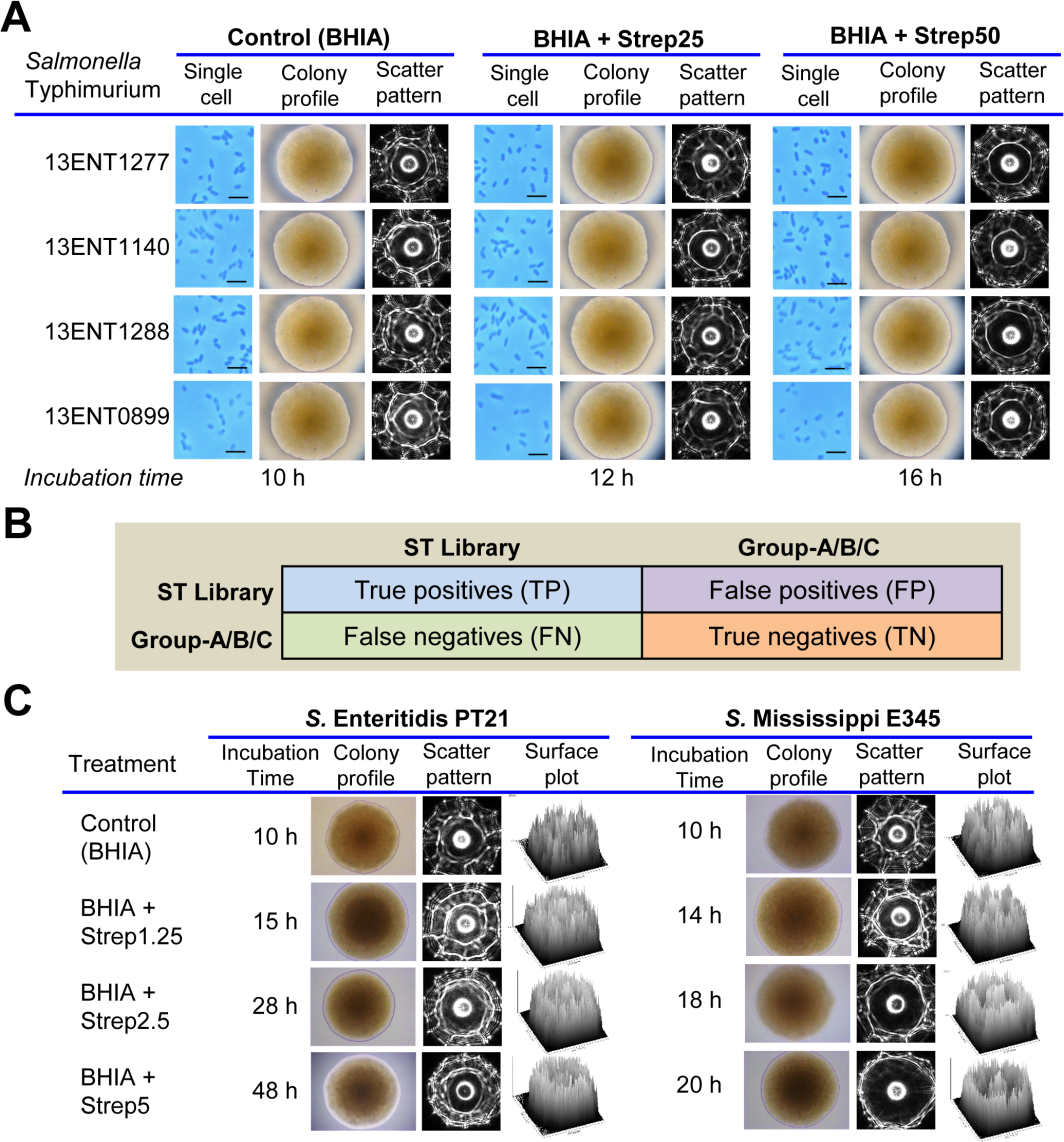
Scatter pattern of *S*. Typhimurium in the presence and absence of streptomycin in BHI agar (BHIA) and error matrix used for analyzing the scatter patterns. **(A)** Scatter patterns of *S*. Typhimurium (13ENT1277, 13ENT1140, 13ENT1288, 13ENT0899) colonies grown on BHIA with or without streptomycin (Strep, 25 and 50 μg/mL) for 10–16 h to achieve a desired colony diameter (1.2 ± 0.1 mm). Phase-contrast microscopic images of individual cells of *S*. Typhimurium obtained from the colony grown under different condition at 1000X (scale bar, 5 μm) to compare the effect of streptomycin on cell size. Two independent experiments were performed to obtain at least 60 scatter images (30 images/experiment) for each strain. Colony profile (diameter) was measured at 100X magnification. Scatter images for BHIA (Control), BHIA+Strep25, and BHIA+Strep50 belongs to Group-A, B and C, respectively. **(B)** Error matrix grid (2 x 2) to calculate true negative values described in **Table 1**. The separate groups of scatter images were acquired from: ST-library containing independent control (without antibiotic) data set; Group-A contains colonies grown on BHIA without antibiotic (BHIA); Group-B contains colonies grown on BHIA supplemented with 25 μg/mL streptomycin (BHI+Strep25); Group-C contains colonies grown on BHIA supplemented with 50 μg/mL streptomycin (BHIA+Strep50). The scatter images in the ST-library were matched with the scatter images of other three groups (A, B, and C) separately in 2 x 2 matrix, to calculate the true negatives. **(C)** Colony profile, scatter pattern and surface plots for the streptomycin-sensitive strains *S*. Enteritidis PT21 and *S*. Mississippi E345 grown on BHIA (control) and BHIA supplemented with sub-inhibitory concentrations of streptomycin, 1.25 μg/mL (Strep1.25), 2.5 μg/mL (Strep2.5), and 5 μg/mL (Strep5). Surface plots were constructed for the scatter images using NIH ImageJ software based analysis of non-RGB images for grayscale values (x-, y-axis: 992 pixels; z-axis, indicates pixel intensity at the scale of 0–255). Scatter patterns were captured at definite time when the colony size reached 1.2 ± 0.1 mm diameter after incubation on respective streptomycin concentration on BHIA plates.

To test the effect of streptomycin on the signature scatter pattern, all four strains of *S*. Typhimurium (13ENT1277, 13ENT1140, and 13ENT1288) were grown on BHIA supplemented with and without 25 μg/mL and 50 μg/mL of streptomycin. All four strains produced visually indistinguishable scatter patterns on BHIA plates lacking any antibiotic supplement. However, the colony scatter patterns started to change with increasing concentrations of streptomycin (25 and 50 μg/mL) (**Fig 3A**). In the presence of streptomycin, radial spokes in the scatter pattern were significantly diminished and a ring around the central spot became more prominent in comparison to the colonies grown on plates without any antibiotic. Moreover, *S*. Typhimurium growth was found to be slower on streptomycin containing BHIA plates, requiring about 12–16 h to reach a colony diameter of ~1 mm, compared to the antibiotic devoid control plates, which needed around 10 h to form 1 mm diameter colony. We ruled out the possibility of using small colony variants (SCV) of *Salmonella enterica* [22,38], as the *S*. Typhimurium cultures grown in streptomycin supplemented BHI broth behaved as a control when plated on BHI agar without any streptomycin and reached the colony diameter of ~1 mm after 10 h of incubation. Similarly, when the primary cultures were not raised in streptomycin supplemented BHI broth, they took >48 h to reach the colony size of ~1 mm diameter. Furthermore, we also did not observe any significant difference in the shape or size of individual bacterial cells, when grown in the presence of streptomycin on BHIA for 12–16 h (**Fig 3A**).

Differences in the scatter pattern of *S*. Typhimurium colonies of each strain grown on BHIA plates with or without streptomycin were calculated using the image classifier [29] and represented as true negatives after comparing the images (Group-A, B, and C) with the ST-library (**Fig 3B**). The percentage of true negative values in all four strains of control group (Group-A, grown in BHIA without antibiotic) was 18.5 ± 5.5%–22.5 ± 7.5%. While the true negative values for strains in BHIA containing S25 (Group-B) were 73.4 ± 7.3%–80.7 ± 5.6%, and for strains with BHIA containing S50 (Group-C) were 90.9 ± 5.1%–95.3 ± 4.6% (**Table 1**). Statistical analysis revealed that the true negative values for control (Group-A) are significantly different (*p* < 0.05) than the Group-B and Group-C (i.e., scatter images from streptomycin containing plates). Minor true negative values observed in control samples (Group-A) are expected since the data set (scatter images) represent scatter patterns (60 scatter images/sample) from two independent experiments, and indicate differences in scatter pattern within the control group. The higher true negative values for scatter pattern obtained from BHIA supplemented with streptomycin (group-B and group-C) indicate a greater difference in the scatter patterns compared to control group-A. This demonstrates the existence of quantitative difference or uniqueness in the scatter images after treatment with the streptomycin compared to untreated control (**Table 1**).

**Table 1:** Differences in the scatter patterns calculated in terms of true negative after *S*. *enterica* serovar Typhimurium colonies grown in the presence and absence (control) of different concentration of streptomycin.

*S*. Typhimurium	% True negative in error/confusion matrix^a^		
	**Control (BHIA) (Group-A)**	**BHIA + Strep25 (Group-B)**	**BHIA + Strep50 (Group-C)**
13ENT1277	20.5 ± 9.5^A^	78.7 ± 6.0^B^	92.8 ± 3.3^C^
13ENT1140	25.5 ± 8.5^A^	73.4 ± 7.3^B^	95.3 ± 4.6^C^
13ENT1288	18.5 ± 5.5^A^	78.7 ± 3.1^B^	90.9 ± 5.1^C^
13ENT0899	22.5 ± 7.5^A^	80.7 ± 5.6^B^	93.8 ± 6.0^C^

^a^Brain-heart infusion agar (BHIA) supplemented with streptomycin 25 μg/mL (BHIA+Strep25), or 50 μg/mL (BHI+Strep50). Values in a row marked with alphabets (A, B, C) indicate significant difference at *p* < 0.05.

Scatter patterns of streptomycin-sensitive *S*. Enteritidis PT21 and *S*. Mississippi E345 colonies also generated differential scatter patterns when grown on BHIA supplemented with the sub-inhibitory concentrations (1.25, 2.5, and 5 μg/mL) of streptomycin compared to BHIA without streptomycin **(Fig 3C**). The Zernike moment features (radial spokes) in scatter images of *S*. Enteritidis PT21 were profoundly reduced in a dose-dependent manner compared to the control BHIA plates. Moreover, the overall growth of *S*. Enteritidis PT21 was substantially slower on 2.5 μg/mL and 5 μg/mL of streptomycin than the control without antibiotics, and the colonies took 28 h and 48 h, respectively, to reach the colony size of 1.2 ± 0.1 mm diameter. Scatter patterns of colonies of *S*. Mississippi E345 also revealed dose-dependent variation in the scatter patterns, where the center ring in the scatter pattern of control colonies appeared to shift towards the periphery of the scatter pattern with increasing concentration of streptomycin on BHIA (**Fig 3C).** In general, sub-inhibitory concentration of streptomycin exhibited a profound difference in the scatter patterns of sensitive serovars (Enteritidis and Mississippi) compared to the differences in scatter pattern of the resistant serovar (Typhimurium). ImageJ based surface plot analysis of scatter pattern also corroborated with the observed differences in scatter patterns and revealed a qualitative difference in the pixel intensities of scatter patterns for streptomycin-sensitive (Enteritidis and Mississippi) grown on BHIA with or without streptomycin (**Fig 3C**).

In BARDOT, the red diode laser (635 nm) interacts with millions and billions of bacterial cells present in the colonial form. Factors like, concentration of nutrients and agar, pH, colony density, temperature, humidity, and bacterial genotype influence the growth of bacterial colonies [39]. Moreover, addition of selective agents or antimicrobial supplements also affects colony growth [29,30,36]. Identification of particular metabolic pathways or specific biomolecules in the bacterial cells, which are responsible for producing specific scatter features (such as spokes, rings or textures) is a challenging quest. However, a key to this unsolved problem may be found at the interface of the physical and biological aspects behind the bacterial colony development. Theoretically, various physical attributes such as refractive index, pH, temperature, optical density, and a number of other biological attributes such as structural organization, aspect ratio, cell surface properties, metabolism, extracellular matrix, and genotypes of colonies, could be responsible for the generation of specific features in a scatter pattern [40].

### Gene expression in response to streptomycin

Streptomycin inhibits protein synthesis in bacteria through binding with the 16S rRNA molecule in the 30S ribosomal subunit [41]. The mechanism by which *S*. Typhimurium and other Gram-negatives exhibit streptomycin resistance is diverse. This may include various proteins responsible for modification of the antibiotic with aminoglycoside adenylyltransferase (AadA) [42]; efflux of antibiotic with aminoglycoside efflux pump (*aep*) that includes a common multidrug resistance subunit periplasmic protein AcrA that bridges integral protein in multidrug efflux pump [43]; and mutation in the ribosomal S12 protein, RpsL also known as StrA [44,45].

**Fig 4:**
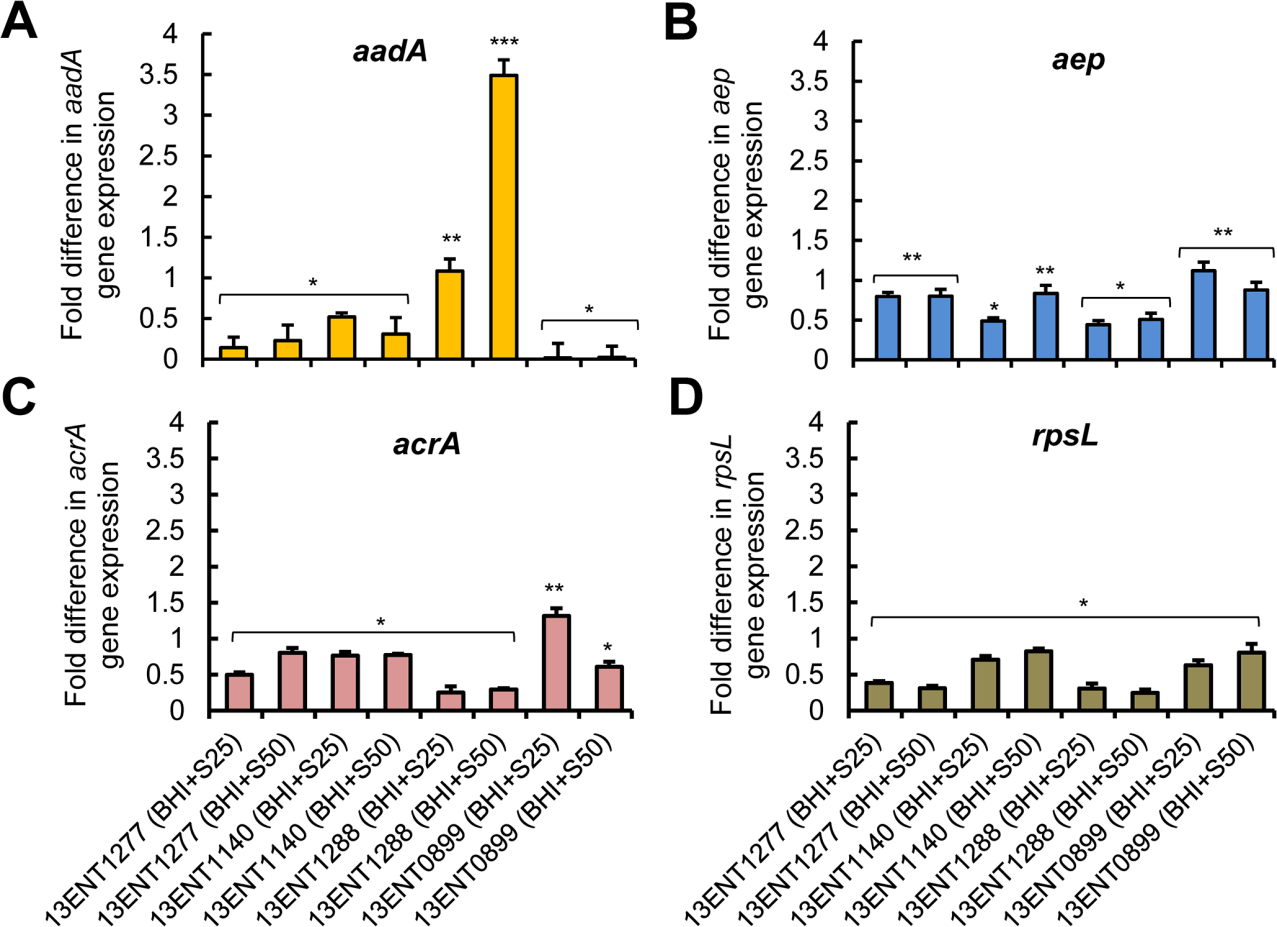
Quantitative reverse transcription PCR (qRT-PCR) to quantify streptomycin resistance-related gene expression in *S*. *enterica* serovars in the presence of streptomycin. Data showing fold difference in the expression of genes coding for (**A**) aminoglycoside adenyltransferase, *aadA*, (**B**) aminoglycoside efflux pump, *aep*, (**C**) multidrug efflux pump subunit, *acrA*, and (**D**) ribosomal S12 protein, *rpsL* (*strA*) after the *S*. Typhimurium cells were grown in BHI broth supplemented with different concentrations (25, 50 μg/mL) of streptomycin (Strep). Bars marked with *, **, *** are significantly different at *p* < 0.05.

The *aadA* gene (786 bases) codes for the AadA enzyme (262 amino acid residues, 29.2 kDa), which modifies the aminoglycoside (streptomycin) [46]. Thus, we examined the effect of streptomycin on *Salmonella* colonies by analyzing the expression of genes coding for AadA, aminoglycoside efflux pump, Aep and its subunit AcrA, and RpsL using quantitative reverse transcription PCR (qRT-PCR). Quantitative RT-PCR results did not reveal any probable dose-dependent changes in the expression of *aadA* gene, except for the strain 13ENT1288 (**Fig 4A**). We also did not observe any significant change (*P* < 0.05) in the expression of *aep*, *acrA*, and *rpsL* in qRT-PCR assay (**Fig 4B, 4C and 4D**). An earlier study by Lang et al. [47] also suggested the absence of observable differential expression of *aadA* gene in *Salmonella* after exposure to streptomycin. Hence, a slight increase in *aadA* gene expression observed in strain 13ENT1288, indicating the strain-dependent AadA-mediated response to streptomycin [38]. Thus, we ruled out any possible involvement of AadA enzyme in observed changes in the scatter pattern of the majority of *S*. Typhimurium colonies. Likewise, the expression of other genes related to streptomycin resistance, *aep* and its subunit *acrA* (**Fig 4B and 4C**), and the ribosomal S12 protein (*rpsL*) (**Fig 4D**) were unaffected in response to streptomycin treatment (25 and 50 μg/mL) suggesting their involvement in scatter pattern changes is uncertain. It is possible, other antibiotic resistance genes such as those encoded in plasmid; provide an opportunity for future investigation.

### Streptomycin induced stress response in *S*. Typhimurium

**Fig 5:**
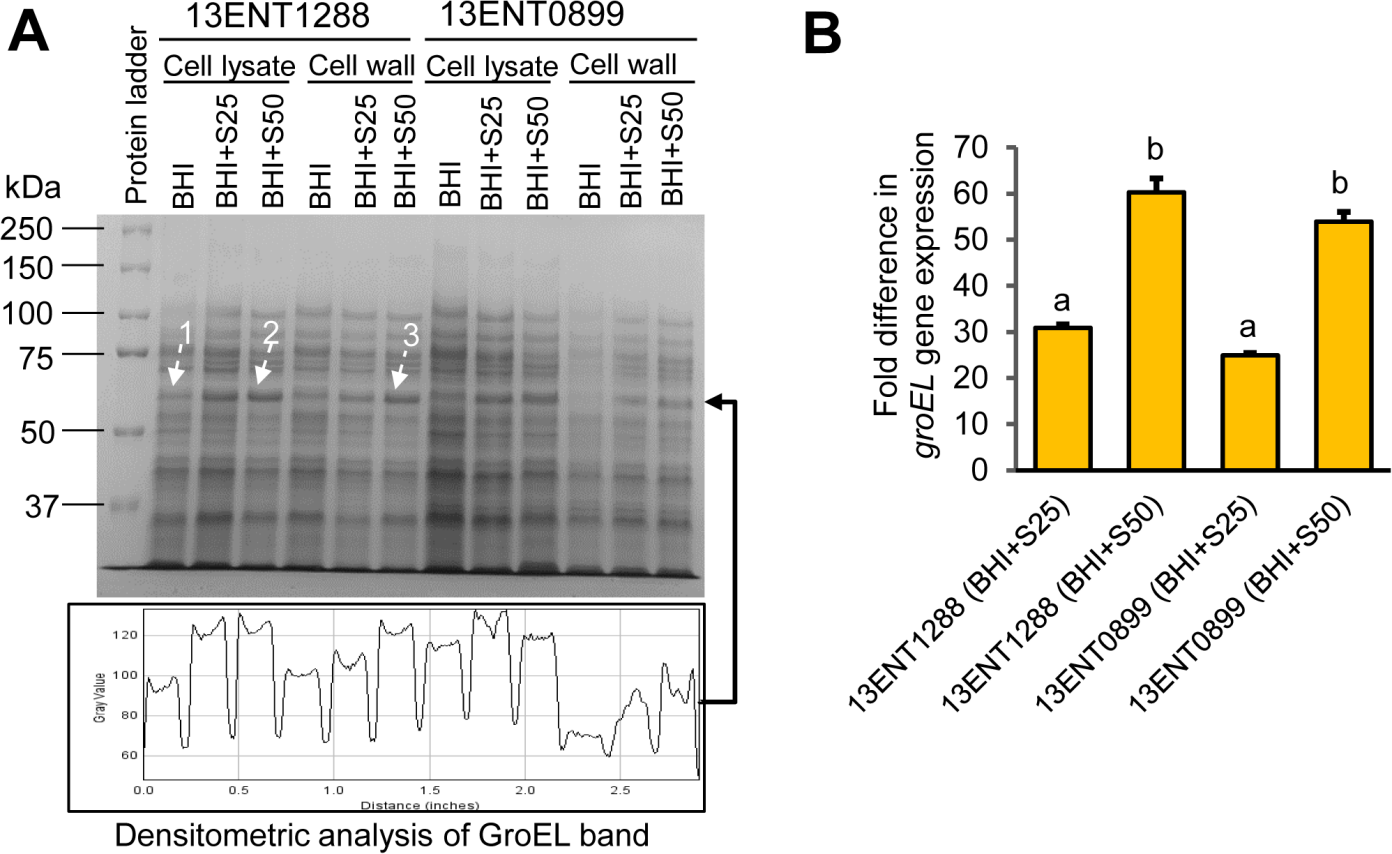
Protein analysis, mass spectrometry and qRT-PCR analysis. **(A)** SDS-PAGE (7.5%-acrylamide)-analysis of protein preparations from whole cell lysate and cell wall/outer membrane. The bottom panel shows quantitative estimation of a protein band (pixel intensity) from the marked lanes using the ImageJ software. Protein bands marked with dashed arrow (1, 2, and 3) were analyzed by MALDI-TOF MS to be GroEL. **(B)** qRT-PCR showing fold difference in the *groEL* gene expression in *S*. Typhimurium strains grown in BHI broth supplemented with streptomycin (25 and 50 μg/mL). Bars marked with the letters (a and b) denote significant difference at *p* < 0.05.

Further, we performed mass spectroscopic analysis to identify possible overexpression of any cellular proteins after exposure to the antibiotic. SDS-polyacrylamide gel electrophoresis (7.5% acrylamide) stained with Coomassie blue showed a differential expression of protein band of ~60 kDa in the cells, representing different treatments and preparations: (1) control cell lysate without antibiotic, (2) cell lysate and (3) cell wall preparations from cells grown in the presence of streptomycin 50 μg/mL (arrow 1, 2 and 3; **Fig 5A**). A densitometry analysis using ImageJ software indicated about 1.1–2-fold increase in the expression of this protein in the presence of streptomycin (**Fig 5A**). MALDI-TOF MS (matrix-assisted laser desorption/ionization-time of flight mass spectroscopy) analysis was performed to identify the protein bands observed in the three lanes (arrow 1, 2 and 3, **Fig 5A**) and all the three protein bands were identified as chaperonin GroEL, showing a 100% protein score confidence index (**Table 2, and S1 Table**). The other two strains, 13ENT1277 and 13ENT1140, also showed a streptomycin-dependent increase in the expression of the same protein (data not shown). The negative aminopeptidase C results obtained from analysis of the protein preparations prior to SDS-PAGE, ruled out the contamination of cytoplasmic proteins with the cell wall/membrane proteins and vice versa (**S1 Fig**) [48].

**Table 2:** MALDI-TOF mass spectrometry based identification of differentially expressed protein bands from *S*. *enterica* serovar Typhimurium after treatment with streptomycin.

Sample^a^	Protein fraction from 13ENT1288^b^	Top ranked species suggested after BLAST analysis^c^	Accession No.	Protein^d^		
				MW (kDa)	PI	Score C.I. (%)
Band-1	Cell lysate (control)	Chaperonin GroEL [*S*. *enterica* serovar Typhi strain E98-0664]	gi|213582265	56.2	4.9	100
Band-2	Cell lysate (Strep50 treated)	chaperonin GroEL [*S*. *enterica* serovar Typhi strain E98-3139]	gi|289830007	59.1	5.0	100
Band-3	Cell wall fraction (Strep50 treated)	molecular chaperone GroEL [*S*. *enterica* serovar Typhi strain CT18]	gi|16763152	57.3	4.9	100

^a^Sample consists of Coommassie blue stained protein band that were sequenced at Applied Biomics (Hayward, CA).

^b^Protein fractions (whole cell lysate and cell wall) were collected from *S*. Typhimurium cell that were grown in BHI broth supplemented with streptomycin at the concentration of 25 μg/mL (Strep25) and 50 μg/mL (Strep50).

^c^Peptide sequence of the band generated by MALDI-TOF MS were compiled and further matched with NCBI protein database. Accession number represents the matched protein.

^d^Represent properties of identified protein; MW: molecular weight; PI: isoelectric point; C.I.: confidence interval.

These results suggest that an increased cell surface expression of GroEL may be responsible for the differential colony scatter patterns when the cells were cultured in the presence of streptomycin. The chaperonin GroEL (536 amino acid residues, 56.2 kDa), encoded by the *groEL* gene (1609 bases), belongs to the Hsp60 family in bacteria. It is required for proper folding of nascent proteins along with other housekeeping functions [49,50]. GroEL chaperonin has also been reported to be localized on the surface of *Lactobacillus johnsonii* strain La1 [51]. In *Acinetobacter baumanii*, an opportunistic nosocomial pathogen, GroEL expression has been shown to be induced upon antibiotic treatment [52].

**Fig 6:**
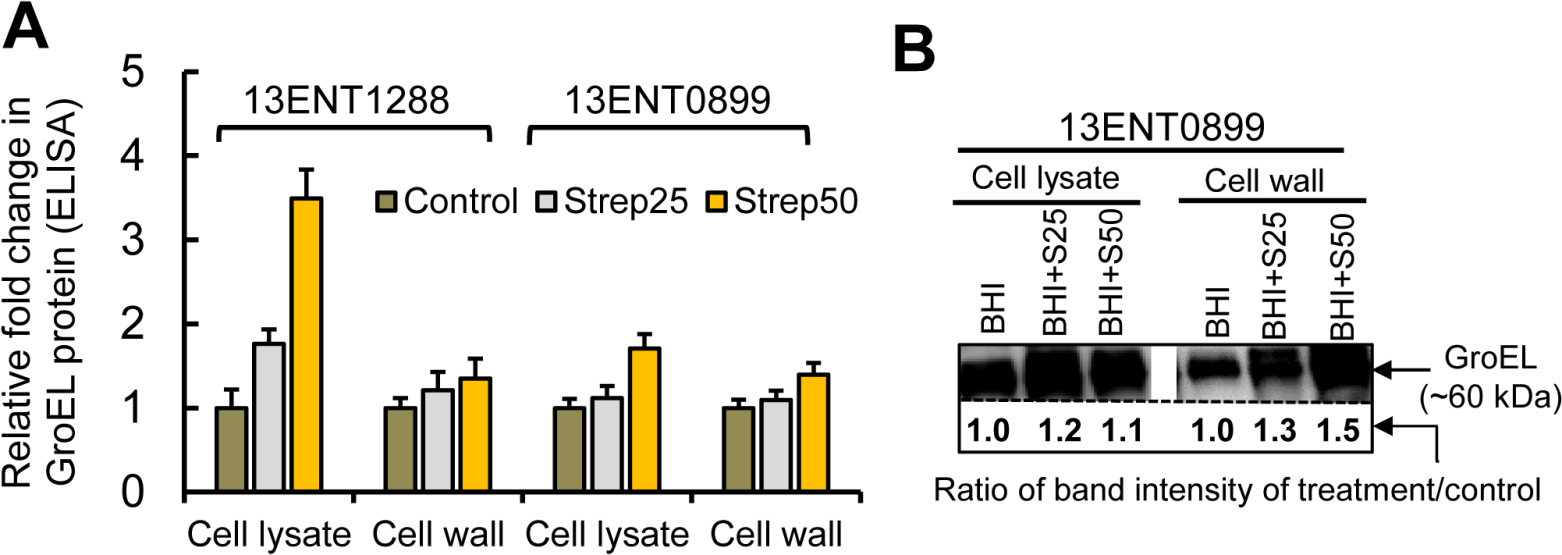
Immunoassays to monitor the streptomycin induced expression of chaperonin GroEL in *S*. Typhimurium. **(A)** ELISA with protein fractions from cell lysate and cell wall/membrane (2 μg/well) of *S*. Typhimurium grown in the presence (25 and 50 μg/mL) or absence of streptomycin was performed. The mouse anti-GroEL mAb (1:2000), HRP-conjugated anti-mouse antibody (1:4000 dilutions; Jackson Immunologicals), and ortho-phenylenediamine (OPD, Sigma-Aldrich) were used in the assay. Data represent, fold change in absorbance values compared to the control. **(B)** Western blot showing over expression of GroEL in the cell fractions after immunoprobing with anti-GroEL mAb (1:1000), HRP-conjugated anti-mouse antibody (1:5000 dilutions; Jackson Immunologicals), and chemiluminescence substrate Lumiglo (Cell signaling, Danvers, MA).

We further verified the expression of *groEL* gene in *Salmonella* using qRT-PCR. qRT-PCR data confirmed an increased *groEL* gene expression, 24.8–30.9-fold and 53.9–60.3-fold in *Salmonella* cells grown in the presence of 25 μg/mL and 50 μg/mL of streptomycin, respectively (**Fig 5B**). Likewise, in enzyme-linked immunosorbent assay (ELISA), anti-GroEL antibody showed 1.1–1.8 and 1.4–3.5 fold increase in absorbance in cell lysate and cell wall/membrane fractions (2 μg/well) from *S*. Typhimurium exposed to 25 and 50 μg/mL of streptomycin, respectively, relative to the untreated cell controls (**Fig 6A**). Western blot analysis also revealed streptomycin (25 and 50 μg/mL) induced GroEL expression in strain 13ENT0899 (**Fig 6B**). In this assay, Coomassie stained gels for total protein (whole cell lysate) and cell wall protein fraction were used as a loading control (**S2 Fig**). GroEL band intensity for Strep25 and Strep 50 samples were quantified using ImageJ software relative to the GroEL band from untreated *S*. Typhimurium samples (control), and data showed 1.1–1.2-fold increase in GroEL band intensity in the presence of 25 μg/mL and 1.3–1.5- fold increase in GroEL band intensity in the presence of 50 μg/mL streptomycin. These data demonstrate that the streptomycin-induced stress caused overexpression of chaperonin GroEL protein. In a previous study, time-dependent Western blot analysis of protein preparation from *A*. *baumanii* cells exposed to streptomycin revealed high induction in heat shock protein, DnaK and GroEL. Earlier studies also reiterated that sub-inhibitory concentration of antibiotic affected cell metabolism [53], and acted as a signaling molecule that triggered specific bacterial response [54] including stress.

We used housekeeping protein, glyceraldehyde-3-phosphate dehydrogenase (GAPDH) as a loading control, but GAPDH expression varied with the streptomycin treatment. Similar observation was also made by earlier investigators [55], who reported that Coomassie stained total proteins are better loading control than the housekeeping proteins. In another study [56], researchers observed that housekeeping proteins are not optimal loading control while studying protein expression in mammalian cells by Western blot and suggested that Coomassie stained total protein to be more reliable.

Voluminous literature available on the mode of action of aminoglycoside conclude that such class of antibiotics corrupts protein translation through binding with the 16S rRNA of 30S ribosomal subunit [41,42,57]. To cope with physiological and environmental stress including response to antibiotics, bacteria exhibit an important protective and homeostatic mechanism at the cellular level with the transient expression of heat shock proteins [58]. An earlier study [59] reported that the exposure of aminoglycoside to bacteria induces chaperonin overexpression that protected the bacterial membrane potential, rescued cell growth, and facilitated survival. In principle, aminoglycosides corrupt the translation process in bacteria leading to protein misfolding [59], and ribosomal stalling and truncated mRNA formation [44]. This induces bacterial system to overexpress Hsp60 chaperonin GroEL in response to the aminoglycoside-induced protein misfolding. Our study also corroborates with previous findings and we observed overexpression of GroEL in response to streptomycin in *S*. Typhimurium through mass spectrometry and immunoassay. In response to the streptomycin treatment *S*. Typhimurium revealed distinguishing colony scatter patterns.

Previous investigators also suggested that the antibiotics may have role as signaling molecules in bacteria [6,54], and when used at sub-inhibitory concentrations, they could promote transcriptional activation of various genes in the bacteria [53]. It has been demonstrated that the sub-inhibitory concentration of antibiotics such as gentamicin and erythromycin targeting protein synthesis affect the transcription of genes involved in transport/binding, metabolism of carbohydrates and amino acids, ribosomal protein synthesis, and purine-pyrimidine biosynthesis [53]. Our study provides avenues to correlate the effect of specific antibiotic-induced metabolic response with the phenotypic scatter pattern of colonies.

BARDOT being an interdisciplinary research tool, will help researchers to understand the intriguing phenomenon occurring at the interface of biology and physics [36,60], and to advance our knowledge of biophysical properties of microbial response to stress or inhibitors for which published literatures are scarce. So far, BARDOT has been explored as a detection and screening tool for pathogens, but in this study, it is used to observe and understand the effect of antibiotic on the colonial form of bacteria, which generally behave as a multicellular entity for intercellular cooperation to cope with stressors and environmental cues [6,40].

## Conclusions

We conclude that the streptomycin exposure to the resistant and sensitive serovars of *S*. *enterica* generated differentiating scatter pattern on BHIA plates. Mass spectroscopy and immunoassay confirmed that streptomycin induced stress response resulted in overexpression of the chaperonin GroEL, which possibly contributed to the observed differences in the optical scatter patterns of the *S*. Typhimurium colonies. Aminoglycoside adenylyltransferase (AadA), aminoglycoside efflux pump (Aep), multidrug resistance subunit AcrA, and ribosomal protein S12 (RpsL), involved in streptomycin resistance in *S*. *enterica* was not upregulated in the presence of streptomycin indicating possible lack of involvement in observed variation in colony scatter patterns. A major point to emphasize is that antibiotic treatment may render pleiotropic effect(s) on the metabolism of bacteria and thus, it would be challenging to single out any specific biomolecule/metabolic pathway responsible for the observed changes in the colony scatter patterns upon exposure to streptomycin. To the best of our knowledge, this is the first report where optical biosensor along with molecular methods were used to monitor and understand the effect of antibiotic-induced stress in bacteria in a colonial form. This report demonstrates the feasibility of a label-free optical biosensor in studying antibiotic-induced stress response in bacteria through colony scatter signatures.

## Materials and Methods

### Clinical isolates, growth media, and chemicals

The top 20 *S*. *enterica* serovars including the *S*. *enterica* serovar Typhimurium strains (13ENT1277, 13ENT1140, 13ENT1288, and 13ENT0899) associated with human-outbreak were obtained from the Indiana State Department of Health (ISDH), Indianapolis, IN, USA. The primary cultures were grown in BHI broth directly from the frozen glycerol stocks stored at -80°C. The dehydrated BHI was purchased from Acumedia (Neogen, Lansing, MI, USA); and nutrient broth (NB), Luria-Bertani (LB) broth, and tryptic soy broth (TSB) were purchased from Becton Dickinson (Sparks, MD, USA). The aminoglycoside, streptomycin sulphate antibiotic (MW: 1457.38; activity: 760 μg/mg) was purchased from Amresco (Solon, OH, USA), and dissolved in ultrapure water at a concentration of 100 mg/mL.

### MIC, optimal media and treatment with streptomycin

The MIC of streptomycin for *S*. *enterica* serovars was determined using micro-titer plate dilution method after taking spectrophotometric absorbance measurements at 595 nm [37] with micro-titer plate reader (Benchmark, BioRad). The MIC of streptomycin was tested with 0–2000 μg/mL concentration of antibiotic. An inoculum of around 10^6^ cells of each strain resuspended in LB broth was added in 200 μL reaction volume and the plates were incubated at 37°C and 70 rpm for 24 h. To find the streptomycin-sensitive strains from the top 20 human-origin *Salmonella* serovars, the assay was performed in BHI broth supplemented with 100 μg/mL streptomycin (Strep100).

To select best media, initially, four agar media (BHIA, LBA, NA, and TSA) were tested to determine which medium would produce the highest scatter features such as Zernike moment and Haralick texture for *Salmonella* colonies [29]. Briefly, the freshly grown primary broth cultures (BHI broth) were serially (10-fold) diluted in 0.1 M phosphate buffered saline, pH 7.4 (PBS), and plated onto BHIA, LBA, NA, and TSA and incubated at 37°C for 12–16 h or until the colony diameter reached to 1.2 ± 0.1 mm. To build the *S*. Typhimurium (ST) library on BHIA, 250 scatter patterns for all the four strains of *S*. Typhimurium were generated from two independent experiments (60 images/strain).

To observe the effect of streptomycin on the colony scatter patterns, cultures were first raised in BHI broth supplemented with sub-inhibitory concentration of streptomycin; 0, 25 and, 50 μg/mL for *S*. Typhimurium; and streptomycin; 0, 5 and 10 μg/mL for *S*. Enteritidis. The cultures were then plated on BHIA containing respective sub-inhibitory concentrations of streptomycin; 0, 25 and, 50 μg/mL for *S*. Typhimurium; and streptomycin concentration (0, 1.25, 2.5, and 5 μg/mL) for *S*. Enteritidis and *S*. Mississippi and incubated at 37°C for 10–20 h or until the colonies reached to 1.2 ± 0.1 mm diameter. Again, BARDOT was used to capture scatter patterns of colonies. The diameter of bacterial colonies and the size of individual cells were measured under a phase-contrast microscope (Leica, Wetzlar, Germany) equipped with Spot software (Sterling Heights, MI) and Leica Application Suite version 4.2.0 (Leica Microsystems, Switzerland) at 100x and 1000x magnifications, respectively. The ST- library was used to demonstrate the effect of streptomycin on *S*. Typhimurium colony scatter patterns [29].

### Instrumentation and image analysis

A schematic of BARDOT based acquisition of scatter patterns of colonies on agar plate is presented in **Fig 1A** and the details of the instrumentation have been described before [27,30]. Briefly, Petri-plate containing colonies was placed in the plate-holder and the laser beam (635 nm) was then passed through the center of each colony [27] generating scatter images for each in seconds. The scatter images are then processed and are analyzed using built-in image analysis software [29,61]. Scatter images based surface plots were also constructed using ImageJ image analysis software [62]. A total of 1486 scatter images obtained from two independent experiments (240 for media optimization; 250 for ST Library, 720 for colonies grown on BHIA with and without streptomycin, and 276 for streptomycin-sensitive serovars) were used in this study.

The difference between the scatter patterns of *S*. Typhimurium colonies grown in the presence or absence of streptomycin was calculated in terms of true negative values **(Fig 3B)** [63] using the image classifier [29]. To calculate the percentage true negatives, scatter images of experimental group (Group-A, Group-B, Group-C) were compared with the ST library on BHIA devoid of streptomycin in a 2 x 2 matrix **(Fig 3B)**. Experimental groups represent scatter images of colonies obtained from BHIA control plate without antibiotic (Group-A), BHIA supplemented with 25 μg/mL streptomycin (Group-B), and BHIA supplemented with 50 μg/mL streptomycin (Group-C). The higher true negative values indicate a greater difference in the scatter patterns of the experimental groups from the ST library. The ST library on BHIA represents four separate groups for each strains and consisted of 250 scatter patterns for all the four strains of *S*. Typhimurium (60 images/strain) that were generated from two independent experiments. The image analysis based comparison between scatter images of ST library and Group-A/B/C in 2x2 matrix, demonstrates how different or unique were the images after treatment with the streptomycin. Data with a high individual score and with a *p* < 0.05 was considered significantly different.

### RNA extraction and qRT-PCR

Total RNA from all *S*. Typhimurium strains (1.89 x 10^9^ ± 0.8 × 10^9^ CFU/mL, equivalent to an OD_600_ of 1.12 ± 0.03 after 12–16 h of growth at 37°C and 180 rpm) was extracted using RNAprotect bacteria reagent and RNeasy minikit (Qiagen, CA, USA). The RNA concentrations were estimated with NanoDrop 2000C (Thermo Scientific, Franklin, MA), and it was in the range of 162.9–977.5 ng/μL. In a 20 μL reaction volume, 2 μg of RNA was used to synthesize cDNA using Supercript Vilo cDNA synthesis kit (Life Technologies, Grand Island, NY). Fast SYBR Green master mix (Life Technologies, Grand Island, NY) was used to perform qRT-PCR using Real-Time StepOnePlus (96-well) PCR system (Applied Biosystem, Foster city, CA) following manufacturers protocol. The Primer-BLAST program (http://www.ncbi.nlm.nih.gov/tools/primer-blast/) was used to design *S*. *enterica*-specific primers for following genes involved in antibiotic resistance and housekeeping function: (a) aminoglycoside adenyltransferase (*aadA*), aminoglycoside efflux pump; (c) multidrug efflux subunit, *acrA*, (d) ribosomal protein S12 (*rpsL*), also known as StrA; and (e) heat shock protein 60, chaperonin GroEL (*groEL*). Eubacterial primers specific for housekeeping gene 16S rRNA (endogenous control) were obtained from previous study [64,65]. The primer sequences are listed in **S2 Table.** The primer sequences are listed in **S2 Table**. The primer specificity was validated using genomic DNA from the strains of *S*. Typhimurium (four strains), *Klebsiella pneumoniae* (4 strains), *A*. *baumanii* (2 strains), *Staphylococcus aureus* (6 strains), *E*. *coli* O157:H7, *Enterococcus faecalis*, *Enterococcus faecium*, *E*. *coli* O157:H7, and *L*. *monocytogenes*. The fold difference in the expression *of* antibiotic resistance and housekeeping genes in *S*. Typhimurium, after growing in different concentrations of streptomycin (0, 25, and 50 μg/mL), was calculated from the relative standard curve obtained using the following equation:

$$Fold\;difference\;in\;target=\frac{Target\;in\;test\;sample}{EC\;in\;test\;sample}x\frac{EC\;in\;calibrator\;sample}{Target\;in\;calibrator\;sample}$$

where, “EC” is the endogenous control (16S rRNA gene), “test sample” is streptomycin treated sample, and “calibrator” is an untreated sample (Life Technologies, Applied Biosystems, NY, USA).

### SDS-PAGE, mass spectroscopy, ELISA and Western blot analysis

For protein analysis, sodium dodecyl sulphate-polyacrylamide gel electrophoresis (SDS-PAGE, 7.5% acrylamide) was performed to identify overexpression of any cellular proteins after exposure to the antibiotic. SDS-PAGE gel was stained with Coomassie blue R250. Whole cell lysate and cell wall/outer membrane protein fractions were prepared separately [48] for 13ENT1288 and 13ENT0899, which were grown in different concentrations of streptomycin (0, 25, 50 μg/mL). The protein fractions were extracted from final cell concentration of 1.89 ± 0.8 × 10^9^ CFU/mL, which was equivalent to an OD_600_ of 1.12 ± 0.03 after 12–16 h of growth at 37°C and 180 rpm. To rule out the contamination of cytoplasmic proteins with the cell wall/outer membrane proteins and vice versa, a standard aminopeptidase C assay was performed [48]. ImageJ software (NIH, Bethesda, MD) was used to measure the density of each protein band. Select protein bands were excised from the stained SDS-PAGE gel and were analyzed using MALDI-TOF mass spectroscopy at Applied Biomics (Hayward, CA, USA). We also performed immunoassays to confirm protein band specificity in SDS-PAGE as a GroEL protein and its expression levels under different treatments using anti-GroEL mAb (Enzo Life Sciences, NY, USA) as described [66]. Although the GroEL antibody was raised against *E*. *coli* GroEL (Enzo Life Sciences), it was expected to react also with *Salmonella* GroEL because GroEL protein from both the bacteria shares high sequence similarity (98.9%).

### Statistical analysis

Statistical analysis was performed using one-way ANOVA (Minitab 16 software) to measure significant difference at *p* < 0.05 with a high individual score. Scatter patterns of each strain of *S*. Typhimurium was obtained from three independent experiments.

## Supporting Information

S1 Fig Aminopeptidase C (pepC) assay to verify contamination of cytoplasmic protein in the cell wall/outer membrane protein fractions of *S*. *enterica* serovar Typhimurium. The extracts were prepared from *S*. Typhimurium 13ENT1288 and 13ENT0899 cells grown in BHI broth in the presence of 25 μg/mL (S25) and 50 μg/mL (S50) of streptomycin and in the absence of streptomycin (Control, CTRL). Absorbance value were recorded after 10 min of incubation at room temperature in a microtiter plate [48].
(TIF)

S2 Fig Coomassie stained SDS-PAGE gel showing total protein (whole cell lysate) and cell wall protein fractions extracted from *S*. Typhimurium 13ENT0899 as the loading control for Western blots, relevant to Fig 6B
(TIF)

S1 Table Mass spectrometry report for the identification of streptomycin-induced protein band as chaperonin GroEL. (XLS)

S2 Table List and sequences of primers used this study. (DOCX)

S1 Video A short video demonstrating the real-time acquisition of scatter images using BARDOT. Video credit: Atul K. Singh and Arun K. Bhunia, Department of Food Science, Purdue University.
(MP4)
