# Supplementary figures and images for: Streptomycin Induced Stress Response in Salmonella enterica Serovar Typhimurium Shows Distinct Colony Scatter Signature
Source: PLoS One. 2015 Aug 7;10(8):e0135035. doi: 10.1371/journal.pone.0135035 (PMC4529181; doi:10.1371/journal.pone.0135035)

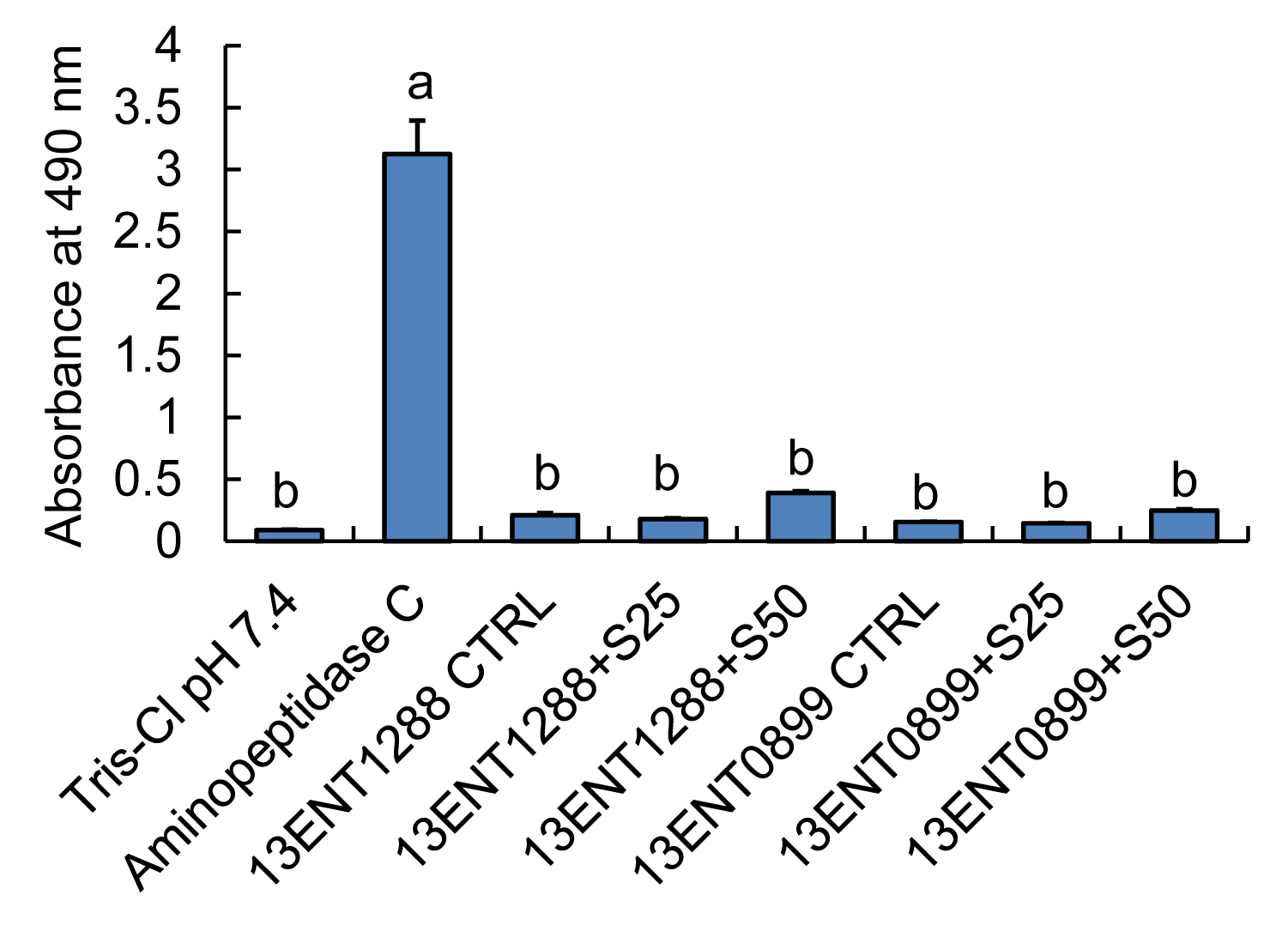

Supplement: S1 Fig — The extracts were prepared from S. Typhimurium 13ENT1288 and 13ENT0899 cells grown in BHI broth in the presence of 25 μg/mL (S25) and 50 μg/mL (S50) of streptomycin and in the absence of streptomycin (Control, CTRL). Absorbance value were recorded after 10 min of incubation at room temperature in a microtiter plate [48]. (TIF) [file pone.0135035.s001.tif]

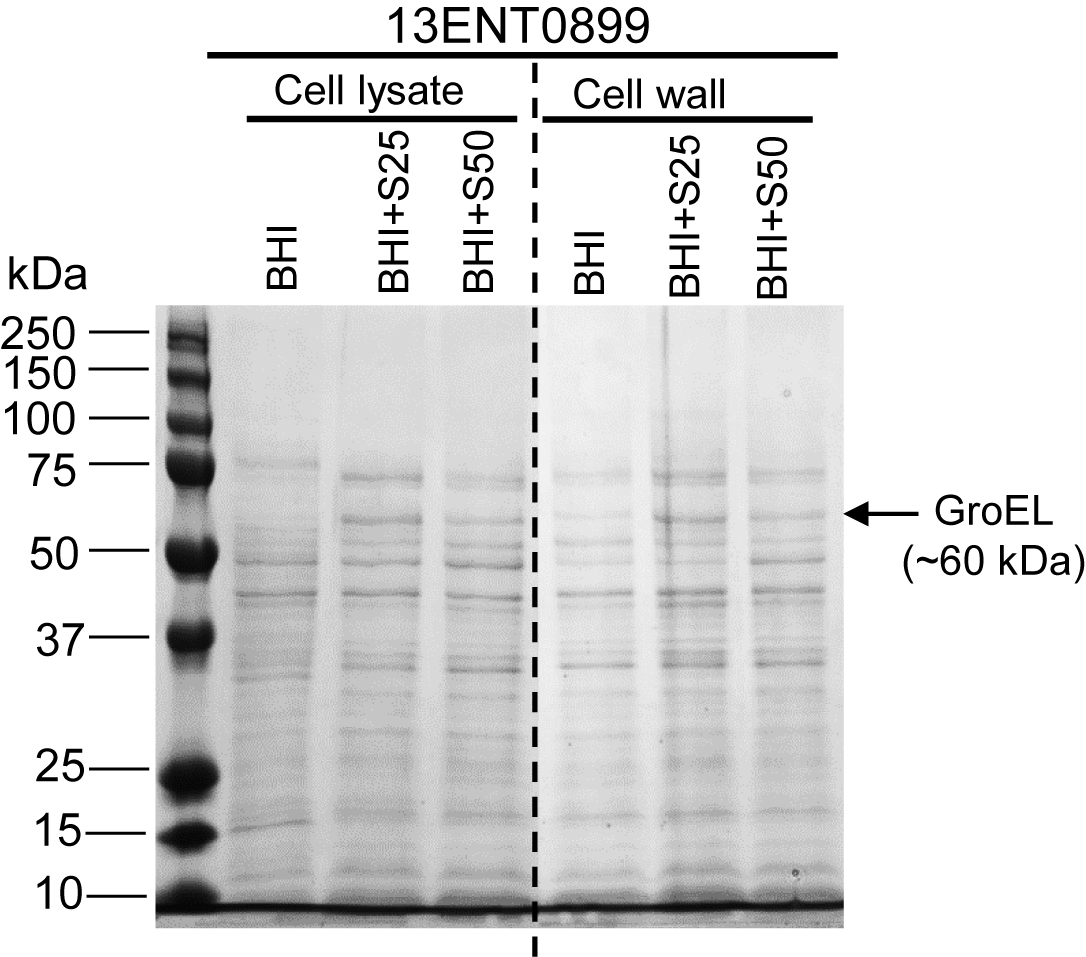

Supplement: S2 Fig — (TIF) [file pone.0135035.s002.tif]
