# Supplementary material for: Streptomycin Induced Stress Response in Salmonella enterica Serovar Typhimurium Shows Distinct Colony Scatter Signature
Source: PLoS One. 2015 Aug 7;10(8):e0135035. doi: 10.1371/journal.pone.0135035 (PMC4529181; doi:10.1371/journal.pone.0135035)
Supplement: S2 Table — (DOCX) [file pone.0135035.s004.docx]

**Table S2.** List and sequences of primers used in this study.

| **Primer sequence (3’-5’)** | **Target Organism (Accession No.)** | **Target gene coding for protein** | **Function** | **Product size (bp)** | **Ref.** |
| --- | --- | --- | --- | --- | --- |
| **aadA(q)-F:** GGAATTGCTGGCGCTTTCAT  **aadA(q)-R:**  GCATTTCCCTGGAAGGTGGA | *S.* Typhimurium LT2 (AE006468) | Aminoglycoside adenyltransferase (AadA) | Antibiotic resistance | 119 | This study |
| **qAEP-F:** GTACGCAAAACGGGCGATAC  **qAEP-R:**  TCGGCGATATCCTGCTTGTC | *S.* Typhimurium LT2 (AE006468) | Aminoglycoside efflux pump | Antibiotic resistance | 83 | This study |
| **qAcrA-F:** TGACCGTTCTGTACCAGTGC  **qAcrA-R:**  AGCAGGAATACGATCAGGCG | *S.* Typhimurium LT2 (AE006468) | Multidrug efflux pump subunit, AcrA | Antibiotic resistance | 178 | This study |
| **qRpsL-F:** ACGAACGGTGTGGTAACGAA  **qRpsL-R:**  CATCGGTGGTGAAGGTCACA | *S.* Typhimurium LT2 (AE006468) | Ribosomal protein S12 (RpsL) | Housekee-ping | 100 | This study |
| **qGroEL-F:** GACCTGAAAGGCCAGAACGA  **qGroEL-R:**  GCGCAGAACGGGTAACTTTG | *S.* Typhi strain E98-0664 (NZ_ CAAU01001059). | Heat shock protein 60, chepronine GroEL | Housekee-ping | 229 | This study |
| **16S 515F:** GTGCCAGCAGCCGCGGTAA  **16S 685R:**  TCTACGCATTTCACCGCTAC | Eubacteria | Non-coding ribosomal RNA gene | - | 170 | [65] |
| **16S-rRNA-F:**  GATGCATAGCCGACCTGAGA  **16S-rRNA-R:**  TGCTCCGTCAGACTTTCGTC | Eubacteria | Non-coding ribosomal RNA gene | - | 143 | [64] |
